# Wastewater analysis for nicotine, cocaine, amphetamines, opioids and cannabis in New York City

**DOI:** 10.1080/20961790.2019.1609388

**Published:** 2019-05-31

**Authors:** Nicole Centazzo, Bonnie-Marie Frederick, Alethea Jacox, Shu-Yuan Cheng, Marta Concheiro-Guisan

**Affiliations:** Department of Sciences, John Jay College of Criminal Justice, City University of New York, New York, NY, USA

**Keywords:** Forensic sciences, forensic toxicology, wastewater-based epidemiology (WBE), nicotine, cocaine, amphetamines, opioids, cannabis, LC-MS/MS

## Abstract

According to current surveys and overdoses data, there is a drug crisis in the USA. Wastewater-based epidemiology (WBE) is an evolving discipline that analyses wastewater samples to detect drugs and metabolites to estimate drug consumption in a certain community. This study demonstrates how drug relative presence could be tracked by testing wastewater, providing real-time results, in different boroughs in New York City throughout 1 year. We developed and fully validated two analytical methods, one for 21 drugs and metabolites, including nicotine, cocaine, amphetamines, opioids and cannabis markers; and another for the normalization factor creatinine. Both methods were performed by liquid chromatography tandem mass spectrometry (LC-MS/MS) using positive electrospray ionization, achieving a limit of quantification of 5–10 ng/L for drugs and metabolites, and 0.01 mg/L for creatinine. These methods were applied to 48 one-time grab wastewater samples collected from six wastewater treatment plants in New York City (Manhattan, The Bronx, Queens and Brooklyn), eight different times throughout 2016, before and after major holidays, including Memorial Day, 4th of July, Labour Day and New Year’s. In this study, the drug group normalized concentrations present in the wastewater samples, in decreasing order, were cocaine, nicotine, opioids, cannabis and amphetamines. When looking at individual compounds, the one with the highest normalized concentration was benzoylecgonine (BE), followed by cotinine, morphine and 11-*nor*-9-carboxy-tetrahydrocannabinol (THCCOOH). To estimate community use, these concentrations were multiplied by the corresponding correction factor, and the most present were THCCOOH, followed by BE, cotinine and morphine. When comparing the treatment plants by drug group (nicotine, cocaine, amphetamines, opioids and cannabis), samples collected from The Bronx had the highest normalized concentrations for nicotine, cocaine and opioids; The Bronx and Manhattan for cannabis; and Manhattan and Queens for amphetamines. In most of the cases, no effect due to holiday was observed. This study provides the first snapshot of drug use in New York City and how that changes between key calendar dates employing wastewater analysis.

## Introduction

In recent years, the abuse of both licit and illicit drugs has significantly grown in the USA. The number of reported overdose cases is steadily increasing, which is of grave concern for healthcare providers and policymakers. According to the 2016 National Survey on Drug Use and Health (NSDUH), it was estimated that 28.6 million individuals aged 12 and older were illicit drug users in the month before the survey was conducted [1]. The number of marijuana users was at its highest in comparison to the data from 2002 to 2015; 24.0 million individuals aged 12 and over reported that they were current users. Almost 2 million individuals admitted to being users of cocaine within the past month from when the survey was conducted, which is a significant increase from 0.9 million users in 2015, and 0.7 million admitted methamphetamine use. Regarding opioids, 11.8 million people aged 12 or older misused prescription opioids in the past year and almost 1 million were heroin users [1]. More than 60% of deaths by overdose in the USA were from opioids in 2015 [2]. In the case of tobacco, it was estimated in 2016 that 38.7 million adults in the US were current cigarette smokers, which is equivalent to 15.5% of the population. Out of the 15.5%, 76.1% reported that they were daily smokers [3]. The use of other tobacco-related products such as electronic cigarettes (e-cigarettes) keeps expanding especially among teenagers, with more than 2 million US middle and high-school students using e-cigarettes in the past 30 days [4]. These drug abuse trends, which are also a worldwide issue [5], have prompted researchers to find ways of efficient and quick estimation of the amount or relative prevalence of drugs that are being consumed in a particular location.

Although surveys provide great insight into what and to what extent drugs are being consumed by the public, these numbers may not be entirely accurate because they are solely based on what people choose to reveal. In addition, data collection methods often exclude people who are separated from society, for instance, homeless and deviant individuals [6], which further obscures the actual numbers. Another important limitation of the classic survey is the delay between the data collection and the availability of the results of about 1–2 years. In 2001, Daughton [7] was the first to suggest that sewage samples from treatment plants of communities could be a useful tool to determine illicit drug use. Since then, the number of publications in the field of wastewater-based epidemiology (WBE) has been increasing. WBE has been most popular in European countries [8–13], but its applications have been expanded to other parts of the world, such as Turkey [14, 15], China [16], Colombia [17], South Africa [18], Cameroon [19] and Australia [20, 21]. In fact, the European Union (European Monitoring Centre for Drugs and Drug Addiction, EMCDDA) and the Government of Australia conduct interdisciplinary wastewater drug monitoring programs, and publish official reports about the drug use in their geographical areas based on wastewater analysis [22, 23].

Despite being a hot topic in different parts of the world, only a few studies in the USA have been performed. In 2004, Jones-Lepp et al. [24] determined the concentrations of two illicit drugs and several pharmaceuticals collecting effluent samples from three different states (Nevada, Utah and South Carolina) over a relatively short period of time (30 days). In 2008, Batt et al. [25] analysed pharmaceuticals in effluent wastewater in New Mexico and surface water in Ohio. Stamper et al. [26] and Foppe et al. [27] investigated the impact of special events, such as football games and solar eclipse, on drug use through wastewater analysis in communities in Mississippi and Kentucky. Burgard et al. [28] investigated prescription stimulants use on a College Campus. Brewer et al. [29] and Skees et al. [30] employed WBE to study drug use in small communities (20 000–50 000 population) in the Pacific Northwest and Midwestern USA. However, no studies have been performed in large cities and for an extended time period.

In addition to the determination of drug concentrations, the studies on population biomarkers present in wastewater are also increasing [31]. In several studies [28, 29], creatinine, a product of muscle metabolism produced at a relatively constant rate throughout the day, has been employed as a normalization factor to account for population variations among sampling periods. In other instances, creatinine has been addressed to have stability issues in the wastewater system [32].

The main analytical challenge in wastewater analysis has been the development of multi-analyte methods including analytes of different chemical properties (basic *vs*. acidic, hydrophilic *vs*. lipophilic). Cannabis’ active compound, δ-9-tetrahydrocannabinol (THC), and its carboxylic acid metabolite, 11-*nor*-9-carboxy-tetrahydrocannabinol (THCCOOH), are especially challenging due to its acidic and lipophilic properties [33–37]. Recently, Causanilles et al. [38] highlighted the analytical issues related to cannabis analysis in wastewater samples, commonly resulting in an underestimation of its use.

In this study, we developed and validated two sensitive and specific methods in wastewater samples, one multi-analyte method for the determination of nicotine, cocaine, amphetamines, opioids and cannabis markers, and a fast and simple method for the determination of creatinine. The methods were applied to 48 wastewater samples collected from the primary settling pool of six treatment plants from four boroughs in New York City, namely Manhattan, The Bronx, Brooklyn and Queens, throughout 1 year. These preliminary data proved useful for the assessment of relative presence for these particular drug groups in different communities in New York City, the most populous city in the USA.

## Materials and methods

### Chemicals and materials

Standards of cotinine, benzoylecgonine (BE), cocaethylene, methamphetamine, 3,4-methylenedioxymethamphetamine (MDMA), 3,4-methylenedioxyamphetamine (MDA), amphetamine, morphine, codeine, oxymorphone, oxycodone, hydromorphone, hydrocodone, fentanyl, norfentanyl, methadone, 2-ethylidene-1,5-dimethyl-3,3-diphenylpyrrolidine (EDDP), THC and THCCOOH were purchased at concentrations of 1 mg/mL in 1 mL methanol. Cocaine and 6-monoacetylmorphine (6-MAM) were acquired at 1 mg/mL in 1 mL acetonitrile. These standards were purchased from Cerilliant Corp (Round Rock, TX, USA), except morphine (Sigma Aldrich, St. Louis, MO, USA). Deuterated internal standards (cotinine-d_4_, cocaine-d_3_, BE-d_3_, cocaethylene-d_3_, amphetamine-d_5_ methamphetamine-d_5_, MDMA-d_5_, morphine-d_3_, codeine-d_3_, oxymorphone-d_3_, oxycodone-d_3_, hydromorphone-d_3_, hydrocodone-d_3_, fentanyl-d_5_, norfentanyl-d_5_, methadone-d_3_, EDDP-d_3_, THC-d_3_ and THCCOOH-d_3_) were purchased at concentrations of 100 µg/mL in 1 mL methanol from Cerilliant. Cocaine-d_3_ and 6-MAM-d_3_ were bought at 100 µg/mL in acetonitrile from the same manufacturer. Creatinine and creatinine-d_3_ powders were purchased from Toronto Research Chemicals (North York, ON, Canada).

Liquid chromatography-mass spectrometry (LC-MS) grade methanol, acetonitrile, isopropanol and formic acid were purchased from Thermo Fisher Scientific (Waltham, MA, USA). Hydrochloric acid (HCl) 36.5%–38% was from J.T. Baker Chemical Co. (Phillipsburg, NJ, USA). Reagent grade dichloromethane and ammonium hydroxide were purchased from Pharmco-Aaper (Brookfield, CT, USA). Nalgene^TM^ certified wide-mouth amber high-density polyethylene (HDPE) 250 mL bottles, Whatman^TM^ glass microfiber filters (outside diameter 4.7 cm, particle retention 1.6 mm, and thickness 0.26 mm), EMD Millipore all-glass filter holder assembly (1 000 mL), Sarstedt Inc 10 mL sc tubes 16 mm × 100 mm, and 350 µL fused insert vials were acquired from Thermo Fisher Scientific. Strata-X-C 33 µm polymeric strong cation exchange solid phase extraction (SPE) cartridges of 3 mL/60 mg and 6 mL/200 mg were purchased from Phenomenex (Torrance, CA, USA). Filter vials eXtreme/FV^®^ 0.2 µm PES with pre-slit grey cap were from Thomson Instrument Company (Oceanside, CA, USA).

### Calibrator, quality control and internal standard working solutions

Standards were diluted with LC-MS grade methanol at a ratio of 1:10 from the original ampoule to final concentrations of 100 µg/mL. Ten milliliter of standard stock solution mixture was prepared at 1 µg/mL and a serial dilution utilizing a 1:10 dilution factor was performed until a final concentration of 0.001 µg/mL was obtained in methanol. Deuterated internal standards were diluted with LC-MS grade methanol at a ratio of 1:10 from the original ampoule to final concentration of 10 µg/mL. Ten milliliter of internal standard (IS) mixture was prepared at 0.1 µg/mL by 1:10 dilution in methanol. Standard and IS working solutions were stored in amber vials at –20 °C. For creatinine and creatinine-d_3_, a stock solution at 1 mg/mL was prepared dissolving 10 mg powder in 10 mL Milli-Q water (Millipore Co., Billerica, MA, USA). Working solutions were prepared by serial 1:10 dilutions in MilliQ water, and were stored in amber vials at 4 °C. The creatinine standard working solution concentrations ranged from 0.01 to 10 µg/mL, and for creatinine-d_3_ the concentration was 0.1 µg/mL.

### Wastewater samples

**Figure 1.**
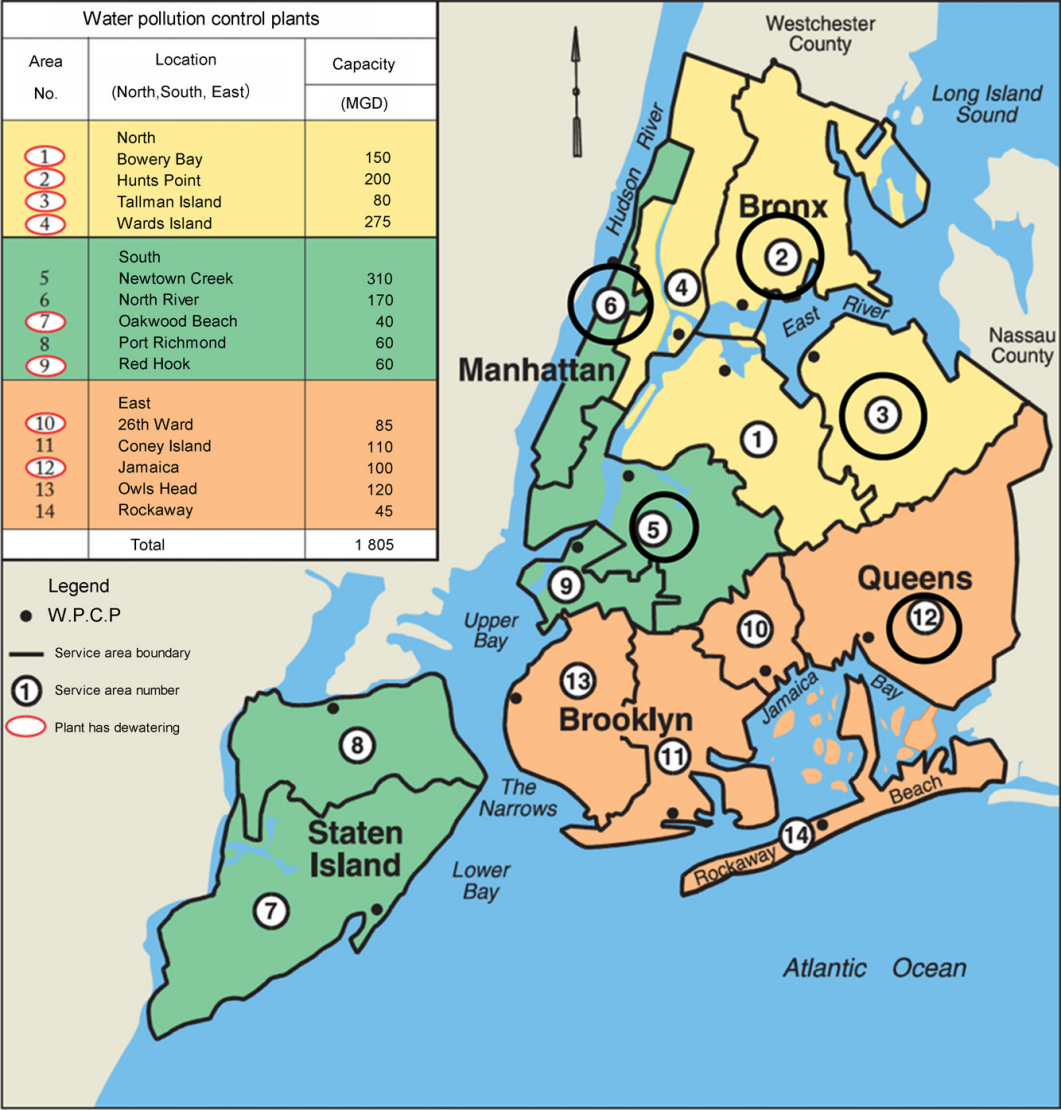
Location and capacity of the wastewater plants in New York City. Circled in black the are wastewater plants included in this study. Information provided by the New York City Department of Environmental Protection (DEP) website (www.nyc.gov/dep). MGD: million gallons per day.

Wastewater samples were collected from wastewater treatment plants (WWTPs) from four municipal boroughs of New York City (Figure 1), namely Manhattan (North River and Newtown Creek-Manhattan pool), The Bronx (Hunts Point), Brooklyn/Queens (Newtown Creek-Brooklyn/Queens pool) and Queens (Tallman Island and Jamaica). North River WWTP has a capacity of 170 million gallons per day (MGD) and serves a population of 588 772 from North River (northern Manhattan at westside of Manhattan above Bank Street); Newton Creek WWTP has a capacity of 310 MGD, and it serves a population of 1 068 012 (south and eastern midtown section of Manhattan, Manhattan pool; northeast section of Brooklyn and western section of Queens, Brooklyn/Queens pool). Hunts Point WWTP has a capacity of 200 MGD and the population served is 684 569 (eastern section of The Bronx). Tallman Island WWTP has a capacity of 80 MGD and the population served is 410 812 (northeast section of Queens). Finally, Jamaica WWTP has a capacity of 100 MGD and it serves a population of 728 123 (southern section of Queens). The type of influent in all these plants is primarily urban residential. These data were retrieved from the New York City Department of Environmental Protection (DEP) website (www.nyc.gov/dep). The choice of these wastewater treatment plants was based on the size of population they serve and study logistics.

According to DEP, after the preliminary treatment to remove large pieces of trash, the wastewater is pumped to the primary settling tanks for 1–2 h. One-time grab samples (in triplicate) from the wastewater plant primary settling pool were performed by DEP authorized personnel. The samples were collected in Nalgene^TM^ certified Wide-mouth amber HDPE 250 mL bottles between 8:00 am and 11:00 am on the collection days. This collection window was based on the DEP personnel’s availability and operating schedule. Sampling was done on days before and after major holidays in 2016 including Memorial Day (27 May, 31 May), 4th of July (1 July, 5 July), Labour Day (2 September, 6 September) and New Year’s (30 December 2016 and 3 January 2017). The samples were stored in coolers and shipped to the laboratory on the same day. Once in the laboratory, the samples were stored at –20 °C until day of analysis.

### Multi-analyte method: sample preparation and solid phase extraction

The method for the simultaneous analysis of nicotine (cotinine), cocaine (BE, cocaethylene, and cocaine), amphetamines (amphetamine, methamphetamine, MDMA and MDA), opioids (6-MAM, morphine, codeine, oxymorphone, oxycodone, hydromorphone, hydrocodone, fentanyl, norfentanyl, methadone and EDDP) and cannabis (THC and THCCOOH) markers was based on a previously published method by our group [39] with modifications. Fifty milliliter aliquots of each authentic wastewater samples were spiked with 25 µL of 0.1 µg/mL IS mixture, containing the deuterated analogues of all the target analytes except MDA. Samples were vacuum filtered using Whatman^TM^ glass microfiber filters. Filtered samples were stored overnight in 100 mL amber glass bottles at 4 °C. Prior to extraction, samples were acidified with 250 µL of HCl.

Quality control samples at 20 and 200 ng/L were prepared using 50 mL of deionized water spiked with 25 µL IS and 100 µL of 0.01 µg/mL (QC 20 ng/L) and 0.1 µg/mL (QC 200 ng/L) of standard stock solutions. LOQs were prepared in 50 mL of deionized water at 5 ng/L and 10 ng/L, using 25 µL and 50 µL of 0.1 µg/mL standard stock solution, respectively. All samples were vacuum filtered and acidified with 250 µL HCl after filtration. To reduce the cost and time of the analytical process, calibrators were prepared in 3 mL of deionized water spiked with the corresponding calibration working solution to match the amounts of 1, 5, 10, 50, 100, 500 and 1 000 ng/L in 50 mL of sample. We added to the calibrators 25 µL of IS mixture at 0.1 µg/mL and 15 µL HCl.

LOQ, low and high QCs and wastewater samples were extracted using Strata-X-C 33 µm 200 mg/6 mL SPE cartridges by Phenomenex. The cartridges were conditioned using 6 mL methanol, 6 mL water and 6 mL 0.1% HCl in water by gravity. Fifty milliliter of the samples were loaded 6 mL at a time. The cartridges were washed using 4 mL water and 4 mL 0.1% HCl in water: acetonitrile (*V*_water_:*V*_acetonitrile_ = 70:30) by gravity. Cartridges were dried for approximately 15 min using vacuum and eluted by gravity with 8 mL of a freshly prepared solution of dichloromethane:isopropanol:ammonium hydroxide (*V*_dichloromethane_:*V*_isopropanol_:*V*_ammoniumhydroxide_=78:20:2).

The calibrators were extracted using Strata-X-C 33 µm 60 mg/3 mL SPE cartridges. Cartridges were conditioned with 3 mL methanol, 3 mL water and 3 mL 0.1% HCl in water. Three milliliter of the samples were loaded and then washed with 2 mL water and 2 mL 0.1% HCl in water:acetonitrile (70:30). Cartridges were dried for 15 min by vacuum and eluted with 4 mL dichloromethane:isopropanol:ammonium hydroxide (78:20:2).

All eluents (wastewater samples, low and high QCs, LOQ, calibrators) were split 50/50 between basic drugs (nicotine, amphetamines, cocaine, opioids) and cannabis, and 100 µL of acidified methanol (*V*_HCl_:*V*_methanol_=1:99) were added only to basic drug labelled tubes. The samples were evaporated at 40 °C using Turbovap^®^ (Biotage, Charlotte, NC, USA) for about 30 min. Basic drug samples were reconstituted with 200 µL 0.1% formic acid in water (mobile phase A). Cannabis samples were reconstituted with 200 µL (*V*_A_:*V*_B_=60:40) mobile phase A and 0.1% formic acid in acetonitrile (mobile phase B).

### Creatinine method: sample preparation, dilution and filtration

One milliliter of wastewater samples was centrifuged (7197 rcf) for 10 min at room temperature. Two hundred microliter of the supernatant were transferred into the shell vial of the Filter Vial™ from Thomson Instrument Company, and fortified with 200 µL of IS, creatinine-d_3_, at 0.1 mg/L. The plunger with filter was slightly inserted into the shell vial, vortexed and then inserted all the way. The filtered sample was directly injected into the LC-MS/MS.

Calibrators were prepared at concentrations 0.01, 0.05, 0.1, 0.5, 1, 5 and 10 mg/L using 200 µL of the corresponding working solution and fortified with 200 µL of IS. QCs at 0.03 mg/L and 3 mg/L were prepared using 200 µL of QC stock solutions (0.03 and 3 mg/L, respectively) and fortified with 200 µL of IS at 0.1 mg/L.

### Instrumental analysis

For the analysis of basic drugs and cannabis, 20 µL each was injected into the LC-MS/MS instrument. The chromatographic separations were carried out on a Nexera UHPLC system (Shimadzu, Columbia, MD, USA). The Nexera UHPLC system consisted of two binary LC-20AD XR high-performance liquid chromatography pumps, online degassing unit (DGU-20A 3 R), cooled autosampler (SIL-20A XR) and an oven (CTO-20AC). The columns, a Kinetex C18 column (2.1 × 100 mm, 1.7 µm) and a C18 guard column, were from Phenomenex. The mobile phase, 0.1% formic acid in water (A) and in acetonitrile (B), was delivered at flow rates of 0.3 mL/min for basic drugs and 0.5 mL/min for cannabis. Column temperature was 30 °C in both separations. For basic drug analysis, the gradient began with holding B at 2% for 1 min then increasing to 30% in 7 min. B increased to 95% from 8 to 10 min and was held for 1 min after which it decreased to 2% from 11 to 11.5 min, and it was held for 2.5 min. Total run time was 14 min. For the analysis of cannabis, the gradient began with B at 40% with an increase to 95% at 3 min and was kept for 1 min. The gradient went back to 40% at 4.5 min, and 40% B was held for 2.5 min. The total run time was 7 min.

The mass spectrometer was a triple quadrupole LCMS-8050 from Shimadzu equipped with electrospray ionization source (ESI). The heating gas and drying gas flows were both at 10 L/min, with a nebulizing gas flow at 2 L/min. The interface temperature was 300 °C and the heat block temperature was 400 °C. All compounds were analysed using ESI in positive ionization mode, and two transitions in multiple reaction monitoring (MRM) mode were acquired for each analyte (Table 1).

**Table 1.** Multiple reaction monitoring (MRM) transitions and the corresponding collision energy (CE, eV) of the analytes included in this study and their internal standards (deuterated analogs).

Compound	Precursor (*m/z*)	Product 1 (*m/z*)	CE (eV)	Product 2 (*m/z*)	CE (eV)
6-MAM	328.0	**165.1**	–40	211.1	–28
6-MAM-d_3_	331.0	165.2	–39	–	–
Amphetamine	136.0	119.2	–22	**91.1**	–14
Amphetamine-d_5_	140.9	93.1	–17	–	–
BE	290.0	**168.2**	–20	105.1	–28
BE-d_3_	293.0	171.2	–21	–	–
Cocaethylene	318.0	**196.2**	–20	82.1	–29
Cocaethylene-d_3_	321.1	199.2	–20	–	–
Cocaine	304.0	**182.2**	–21	82.1	–30
Cocaine-d_3_	306.9	185.3	–22	–	–
Codeine	300.0	**215.1**	–39	165.2	–28
Codeine-d_3_	303.0	165.1	–43	–	–
Cotinine	177.1	**80.2**	–24	53.2	–45
Cotinine-d_3_	180.1	80.1	–25	–	–
Creatinine	114.0	**44.0**	–18	86.0	–16
Creatinine- d_3_	117.0	**47.0**	–21	89.1	–15
EDDP	279.1	**235.2**	–32	220.2	–45
EDDP-d_3_	282.0	235.2	–32	–	–
Fentanyl	337.1	**188.3**	–24	105.2	–39
Fentanyl-d_5_	342.1	188.3	–24	–	–
Hydrocodone	300.0	**199.2**	–30	171.2	–39
Hydrocodone-d_3_	303.0	199.2	–31	–	–
Hydromorphone	286.0	**185.1**	–32	157.2	–44
Hydromorphone-d_3_	289.0	185.2	–32	–	–
MDA	180.1	163.2	–15	**105.2**	–25
MDMA	194.1	**163.1**	–15	105.1	–24
MDMA-d_5_	199.2	165.2	–15	–	–
Methadone	310.0	**105.2**	–26	77.2	–53
Methadone-d_3_	312.9	268.3	–16	–	–
Methamphetamine	150.1	119.2	–15	**91.2**	–21
Methamphetamine-d_5_	155.2	121.2	–16	–	–
Morphine	286.0	**201.2**	–41	165.2	–28
Morphine-d_3_	289.0	181.1	–35	–	–
Norfentanyl	232.9	**84.2**	–19	55.2	–35
Norfentanyl-d_5_	238.1	84.2	–20	–	–
Oxycodone	316.0	**241.2**	–30	212.1	–43
Oxycodone-d_3_	319.1	244.2	–30	–	–
Oxymorphone	302.0	227.2	–28	**198.1**	–48
Oxymorphone-d_3_	305.0	230.2	–30	–	–
THC	315.0	**193.2**	–22	123.1	–33
THC-d_3_	318.1	196.2	–24	–	–
THCCOOH	345.0	**299.3**	–21	193.2	–27
THCCOOH-d_3_	348.0	302.2	–21	–	–

The quantifier ions are in bold. 6-MAM: 6-monoacetylmorphine; BE: benzoylecgonine; EDDP: 2-ethylidene-1,5-dimethyl-3,3-diphenylpyrrolidine; MDA: 3,4-methylenedioxyamphetamine; MDMA: 3,4-methylenedioxymethamphetamine; THC: δ-9-tetrahydrocannabinol; THCCOOH: 11-*nor*-9-carboxy-tetrahydrocannabinol; –: not detected.

In the case of creatinine analysis, the same LC-MS/MS system, Nexera UHPLC and LCMS-8050 from Shimadzu, was employed; 20 µL of the filtered sample were injected into the LC-MS/MS, and the chromatographic separation was performed using a Luna C8 column (2 × 150 mm, 3 µm) (Phenomenex). The mobile phase, 0.1% formic acid in water (A) and in acetonitrile (B), was delivered at flow rate of 0.3 mL/min. The column temperature was 30 °C. The gradient increased from 2% to 15% B in 1.5 min and then to 95% in 2 min, and it was held from 3.5 to 4 min. Then, it decreased to 2% from 4 to 4.5 min and was held until 6 min. The MS source parameters for this method were the same as those used for the basic drugs and cannabis analysis. Creatinine was analysed in positive ESI ionization mode with two MRM transitions being monitored (Table 1).

### Data analysis

The focuses of the quantification analysis were to determine the concentrations of drugs, metabolites and drug groups among the six WWTPs, and to determine whether or not a relationship exists between drug presence and holiday. All drug concentrations were normalized by creatinine concentrations to account for population variations and dilution. In order to determine the normalized values, the ratio between the concentration of a drug or metabolite and the concentration of creatinine was calculated (ng/mg creatinine). The analyte concentration values were converted from ng/mg creatinine to nmol/mmol creatinine for drug group comparison. The compounds were grouped according to their drug family, except when looking at the highest and lowest analyte concentration in all of the WWTPs looking at each compound individually.

To convert the analyte concentrations determined in wastewater to estimates of community use, the normalized concentrations of the individual compounds were multiplied by the correction factor for each drug, when available. These factors for stimulants (cocaine, amphetamine, methamphetamine, MDMA), opioids (codeine, morphine, heroin, fentanyl and methadone), cannabis and nicotine were based on the current literature [37, 40].

One-way ANOVA was used for the comparison of concentrations between different locations and between different holidays. Two-way ANOVA was used to determine whether or not there was a significant difference in concentrations between holidays, locations and holiday and location as a combined effect. To determine whether any significant difference existed, *P*-values that were less than 0.05 were considered significant. In order to perform this statistical analysis, six assumptions were made: (1) the dependent variable was measured at the continuous level; (2) the independent variables consisted each of two or more sub-categories; (3) independence of observation; (4) there were no significant outliers; (5) the data were approximately normally distributed; (6) the variances were homogeneous. These tests were done by using *R* (free software environment for statistical computing and graphics supported by the *R* Foundation for Statistical Computing).

## Results

### Multi-analyte method

The multi-analyte method was validated adapting the Scientific Working Group for Forensic Toxicology (SWGTOX) guidelines [41] to wastewater analysis. Due to the lack of negative wastewater samples for the compounds of interest, the linearity, accuracy and precision were evaluated employing deionized water, and extraction efficiency and matrix effect using river water samples from different locations of the Hudson and East rivers.

Linearity was demonstrated using 7-point calibration curves (*n* = 6) from 5–10 to 1 000 ng/L. Curves were linear with coefficients of determination (*R*^2^) ranging from 0.9818 to 0.9920, and residuals were within ±20%. To determine the accuracy and precision of the method, duplicates of 50 mL QC samples at two concentrations 20 ng/L (low QC) and 200 ng/L (high QC) for each of the six days of analysis were analysed (*n* = 12). The low QC accuracies ranged from 104% to 120% for basic drugs and from 114% to 116% for cannabis. The high QC accuracies were from 99% to 114% for basic drugs and from 104% to 110% for cannabis. With regard to the precision for basic drugs, the range was from 87.1% to 96.8%, while for cannabis the range was from 90.4% to 94.6% when considering the low QCs (20 ng/L). The high QCs have ranges of 88.9%–96.6% for basic drugs and of 82.8%–94.8% for cannabis.

Extraction efficiency and matrix effects were done by fortifying samples before and after the analytical procedure at 20 ng/L. Three negative river water samples were fortified before the extraction and six were fortified after. The extraction efficiencies for basic drugs were between 54.1% and 95.2%, and 7.7% and 22.0% for cannabis. For matrix effects, the percent range was –46.4% to –10.8% for basic drugs and –6.2% to –5.1% for cannabis. The CV for the six river samples ranged from 3.9% to 20.1% for basic drugs and 9.1% to 11.6% for cannabis. These results are summarized in Table 2. Extracted samples were stable in the autosampler for 24 h at 10 °C. No carryover was detected after injections at the upper limit of quantification.

**Table 2.** Linearity, accuracy, precision, matrix effect (low QC, 20 ng/L) and extraction efficiency (low QC, 20 ng/L) of nicotine, amphetamines, cocaine, opioids and cannabis markers included in this study.

Analytes	Linearity (*R*^2^ mean ± SD, *n* = 6)	Accuracy (%, *n* = 12)		Precision (%, *n* = 12)		Matrix effect (%, *n* = 6)	Extraction efficiency (%, *n* = 6)
		QC 20 (ng/L)	QC 200 (ng/L)	QC 20 (ng/L)	QC 200 (ng/L)		
6-MAM	0.9888 ± 0.0172	107	100	89.7	88.9	5.8	75.6
Amphetamine	0.9894 ± 0.6410	114	106	94.7	92.8	–7.4	77.5
BE	0.9865 ± 0.0090	110	100	94.0	93.8	–5.2	95.2
Cocaethylene	0.9900 ± 0.0130	113	103	96.1	93.5	–41.6	71.8
Cocaine	0.9920 ± 0.0106	112	106	93.7	94.6	–46.4	64.8
Codeine	0.9899 ± 0.0075	114	112	92.3	94.9	4.8	77.6
Cotinine	0.9857 ± 0.0385	104	107	90.5	93.2	10.8	73.3
EDDP	0.9916 ± 0.9700	110	99	94.0	91.5	–22.8	54.1
Fentanyl	0.9844 ± 0.0058	115	112	96.8	95.6	–43.8	72.9
Hydrocodone	0.9866 ± 0.0186	104	100	94.2	89.9	–27.0	87.7
Hydromorphone	0.9875 ± 0.0075	105	105	91.7	90.7	–15.2	88.5
MDA	0.9911 ± 0.0017	120	114	93.5	94.3	–3.0	75.3
MDMA	0.9832 ± 0.0144	112	104	93.6	90.5	–19.2	71.4
Methadone	0.9884 ± 0.0024	112	108	95.4	96.6	–23.3	72.3
Methamphetamine	0.9898 ± 0.8590	114	106	95.9	95.2	–14.5	73.6
Morphine	0.9849 ± 0.0143	107	103	89.3	90.7	–16.5	82.5
Norfentanyl	0.9892 ± 0.0148	109	108	95.5	94.5	–13.8	77.8
Oxycodone	0.9864 ± 0.0155	107	99	91.9	91.8	–14.1	79.8
Oxymorphone	0.9888 ± 0.0124	110	109	87.1	89.2	–21.9	87.0
THC	0.9830 ± 0.0292	116	110	90.4	82.8	–6.2	7.7
THCCOOH	0.9882 ± 0.0267	114	104	94.6	94.8	–5.1	22.0

6-MAM: 6-monoacetylmorphine; BE: benzoylecgonine; EDDP: 2-ethylidene-1,5-dimethyl-3,3-diphenylpyrrolidine; MDA: 3,4-methylenedioxyamphetamine; MDMA: 3,4-methylenedioxymethamphetamine; THC: δ-9-tetrahydrocannabinol; THCCOOH: 11-*nor*-9-carboxy-tetrahydrocannabinol.

### Creatinine method

The creatinine method was also validated adapting SWGTOX guidelines [41]. As it happened in the multi-analyte method, due to the lack of negative wastewater samples for creatinine, deionized water was employed in the evaluation of linearity, accuracy, precision and extraction efficiency. Linearity for creatinine was demonstrated using 7-point calibration curves (*n* = 4) from 0.01 to 10 mg/L; *R*^2^ ranged from 0.9997 to 0.9998 (0.99979 ± 0.0009) and residuals were within ±20%. Triplicates of low (0.03 mg/L) and high (3 mg/L) QCs in 4 different days were used to measure the accuracy and precision (*n* = 12). Accuracy was 103% for the low QC and 96% for the high QC. Precisions were calculated by considering the mean for all 4 days of analysis and were 92.8% for low QC and 96% for high QC. Extraction efficiency was evaluated at low and high QCs by analysing triplicate water samples that were both diluted and filtered and those that were only diluted with IS, to evaluate the potential loss due to filtration. The extraction efficiencies were between 95.8% and 98.2%.

In the case of the evaluation of matrix effect, river samples could not be employed as alternative matrix due to the presence of creatinine in those samples. We investigated matrix effect in the surrogate analyte creatinine-d_3_. The matrix effects were evaluated by comparing the creatinine-d_3_ peak areas in QC samples in deionized water (*n* = 18) and in authentic wastewater samples (*n* = 48). The analysis showed ion suppression of –75.7%. The CV was calculated for the authentic samples and it was 39.1% (*n* = 48). The samples were stable for at least 96 h at 4 °C in the filtration vials. No interferences were detected by the presence of the other drugs and metabolites that were analysed in this study.

### Authentic wastewater samples

#### Creatinine

All samples were positive for creatinine in all WWTPs. The highest concentration was detected in Newtown Creek-Brooklyn/Queens (2.68 mg/L) and the lowest concentration in Tallman (0.22 mg/L), with a median of 1.14 mg/L. The results (median, range) for each WWTP were: Hunts Point (The Bronx) 0.81, 0.47–1.37 mg/L; Jamaica (Queens) 1.06, 0.68–1.66 mg/L; Newtown Creek-Manhattan pool 1.19, 0.90–1.61 mg/L; Newtown Creek-Brooklyn/Queens pool 1.64, 1.38–2.68 mg/L; Tallman (Queens) 0.59, 0.22–1.94 mg/L; and North River (northern Manhattan) 1.34, 0.95–1.93 mg/L.

#### Nicotine

**Figure 2.**
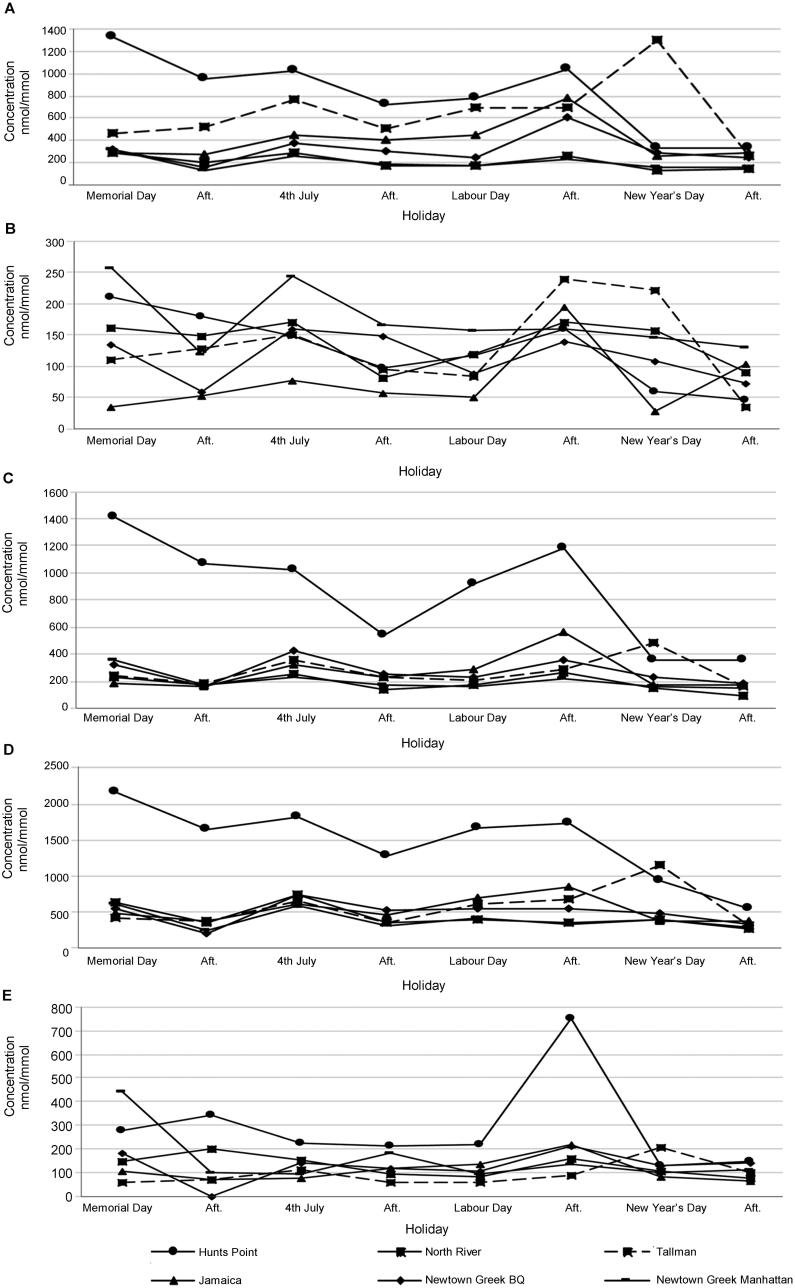
Groups of (A) nicotine, (B) amphetamines, (C) opioids, (D) cocaines, and (E) cannabinoids normalized concentrations (nmol/mmol creatinine) in six wastewater plants in New York City. BQ: Brooklyn/Queens.

The compound analysed to detect nicotine exposure was cotinine, nicotine’s major metabolite. All samples were positive for cotinine. The normalized concentration ranges by location and by collection date are summarized in Tables 3 and 4. The highest concentration (2 075.1 ng/mg) was seen at Hunts Point (The Bronx) before Memorial Day, and the lowest concentration (187.6 ng/mg) was seen at North River (northern Manhattan) before New Year’s. An effect for treatment plant location was observed (*F* = 13.164, *P* = 3.14 × 10^–6^) so that the average concentrations found in Hunts Point were significantly higher than the other treatment plants, except Tallman. No effect due to holidays (*F* = 1.968, *P* = 0.146) and to the interaction of holiday and location (*F* = 1.372, *P* = 0.238) was observed. Cotinine results for all WWTPs and collection times are shown in Figure 2(A) and Supplementary Tables S1–S6.

**Table 3.** Number of positive samples and normalized concentration ranges (ng/mg creatinine) of 21 drugs and metabolites by location at eight different time points throughout 2016.

Analytes	Wastewater plant											
	Hunts Point		North River		Tallman		Jamaica		Newtown Creek-Brooklyn/Queens		Newtown-Creek Manhattan	
	*n*	Range	*n*	Range	*n*	Range	*n*	Range	*n*	Range	*n*	Range
Cotinine	8	507.0–2 075.1	8	187.6–449.6	8	394.2–2 027.9	8	395.1–1 210.4	8	248.1–944.7	8	200.1–495.4
Morphine	8	361.7–1 942.7	8	82.7–356.2	8	236.3–674.8	8	196.9–753.9	8	211.9–596.5	8	163.3–320.9
Oxymorphone	8	48.9–166.5	8	15.3–40.4	8	27.5–108.6	8	27.9–76.5	8	13.1–27.9	8	22.0–52.8
Hydromorphone	8	32.2–108.2	8	4.2–35.8	8	5.3–20.7	8	6.3–16.1	8	4.1–13.0	8	5.1–234.2
Codeine	8	56.0–294.6	8	34.2–104.8	8	33.5–140.4	8	42.6–321.1	8	39.9–99.7	8	31.5–85.4
Oxycodone	8	24.6–107.2	8	7.8–31.5	8	20.5–127.5	8	21.1–50.9	8	6.7–19.4	8	16.7–37.3
6-MAM	7	4.8–25.4	ND	ND	ND	ND	1	9.4	ND	ND	ND	ND
Hydrocodone	4	6.3–8.5	8	3.0–7.4	3	7.4–10.7	7	2.9–11.6	6	1.9–4.0	5	3.0–10.6
Norfentanyl	ND	ND	ND	ND	ND	ND	ND	ND	ND	ND	ND	ND
Fentanyl	ND	ND	ND	ND	ND	ND	ND	ND	ND	ND	1	5.3
EDDP	8	176.0–698.1	8	55.9–142.4	8	49.0–120.7	8	36.4–131.7	8	85.1–226.4	8	88.7–132.5
Methadone	8	91.3–361.2	8	23.4–56.0	8	19.6–54.5	8	17.3–69.2	8	42.0–124.3	8	32.0–81.8
Amphetamine	8	12.9–87.7	8	70.4–174.2	8	22.6–125.7	8	14.2–98.7	8	42.3–123.7	8	109.2–265.1
MDA	ND	ND	5	4.2–26.6	2	12.9–16.1	3	6.6–37.4	7	2.7–24.8	5	6.0–13.5
Methamphetamine	8	15.7–244.4	8	148.7–309.9	8	25.4–340.5	8	17.8–165.8	8	24.8–144.0	8	60.9–141.3
MDMA	1	9.9	8	5.9–97.2	8	9.7–223.8	5	4.7–109.4	8	6.7–48.6	8	7.3–52.3
BE	8	995.1–3 947.2	8	481.4–1 223.9	8	641.5–2 132.9	8	663.0–1 670.6	8	389.6–1 411.6	8	469.5–1 100.3
Cocaine	8	370.5–1 814.8	8	162.3–633.5	8	162.3–829.3	8	224.9–488.1	8	92.4–465.7	8	144.5–457.0
Cocaethylene	8	10.6–57.0	8	9.4–29.0	8	5.8–21.2	8	8.0–17.4	8	6.6–31.7	8	7.6–30.1
THC	ND	ND	8	4.9–30.4	ND	ND	7	3.0–12.1	ND	ND	ND	ND
THCCOOH	8	394.3–2 290.0	8	226.4–571.9	8	172.6–630.8	8	200.0–650.1	8	328.0–650.2	8	277.3–1 354.8

ND: not detected; 6-MAM: 6-monoacetylmorphine; EDDP: 2-ethylidene-1,5-dimethyl-3,3-diphenylpyrrolidine; MDA: 3,4-methylenedioxyamphetamine; MDMA: 3,4-methylenedioxymethamphetamine; BE: benzoylecgonine; THC: δ-9-tetrahydrocannabinol; THCCOOH: 11-*nor*-9-carboxy-tetrahydrocannabinol.

**Table 4.** Number of positive samples and normalized concentration ranges (ng/mg creatinine) of 21 drugs and metabolites by collection date in six wastewater plants in New York City.

Analytes	2016/2017 Collection dates															
	Memorial Day				4th July				Labour Day				New Year’s			
	May 27		May 31		July 1		July 5		September 2		September 6		December 30		January 3	
	*n*	Range	*n*	Range	*n*	Range	*n*	Range	*n*	Range	*n*	Range	*n*	Range	*n*	Range
Cotinine	6	442.7–2 075.1	6	200.1–1 478.9	6	393.0–1 593.0	6	262.3–1 126.2	6	267–1 221.6	6	353.8–1 627.4	6	187.6–507.0	6	208.3–509.8
Morphine	6	215.8–1 942.7	6	184.7–1 402.8	6	226.0–1 271.5	6	151.9–503.0	6	178.2–1 215.1	6	254.2–1 436.9	6	163.3–674.8	6	82.7–409.1
Oxymorphone	6	18.6–166.5	6	13.1–113.5	6	27.9–122.9	6	16.3–75.5	6	14.6–78.3	6	20.1–138.1	6	13.8–108.6	6	14.6–48.9
Hydromorphone	6	6.3–234.2	6	4.2–58.5	6	10.7–53.0	6	4.2–61.2	6	4.6–63.1	6	7.3–108.2	6	6.2–32.2	6	4.1–40.9
Codeine	6	51.7–294.6	6	31.5–191.3	6	83.8–199.2	6	43.9–94.3	6	41.6–176.6	6	67.5–321.1	6	39.6–132.0	6	34.2–110.4
Oxycodone	6	12.6–107.2	6	6.7–66.5	6	19.4–93.4	6	11.3–45.0	6	10.5–44.4	6	11.3–85.8	6	8.4–127.5	6	7.8–34.6
6-MAM	ND	ND	1	18.6	1	8.0	1	25.4	1	4.8	2	7.1–9.4	1	16.1	1	5.2
Hydrocodone	6	3.7–7.4	5	1.9–8.5	5	5.0–10.7	4	3.5–6.1	3	2.3–7.3	5	4.0–11.6	3	2.9–3.1	2	3.0–10.6
Norfentanyl	ND	ND	ND	ND	ND	ND	ND	ND	ND	ND	ND	ND	ND	ND	ND	ND
Fentanyl	ND	ND	ND	ND	ND	ND	0	5.3	ND	ND	ND	ND	ND	ND	ND	ND
EDDP	6	51.6–655.9	6	49.0–583.0	6	81.0–558.0	6	59.9–396.0	6	49.0–505.2	6	61.5–698.1	6	60.9–229.4	6	36.4–176.1
Methadone	6	19.7–361.2	6	19.6–296.1	6	47.2–290.5	6	23.4–192.8	6	28.2–259.0	6	29.7–323.8	6	30.2–135.1	6	17.3–91.3
Amphetamine	6	25.7–265.1	6	40.2–125.2	6	49.9–260.9	6	32.8–156.6	6	27.8–167.1	6	50.0–128.6	6	12.9–125.7	6	14.3–117.3
MDA	1	2.7	3	3.6–13.4	1	4.7	5	4.2–24.8	1	10.0	5	10.0–37.4	3	13.5–26.6	4	5.8–10.8
Methamphetamine	6	17.8	6	24.8–309.9	6	68.6–283.6	6	34.1–228.4	6	27.3–216.9	6	53.2–318.6	6	28.0–340.5	6	15.7–165.8
MDMA	5	5.9–58.4	5	10.1–125.1	4	11.1–54.8	5	13.8–62.2	4	7.3–46.5	5	35.9–223.8	5	4.7–167.6	5	9.7–92.4
BE	6	700.9–3 947.2	6	389.6–3 079.0	6	1 016.9–2 886.1	6	604.9–2 526.4	6	752.7–3 248.7	6	644.8–3 346.8	6	655.1–2 132.9	6	481.4–995.1
Cocaine	6	219.6–1 613.4	6	92.4–1 130.0	6	446.4–1 814.8	6	184.2–761.1	6	225.8–1 041.1	6	198.1–1 118.1	6	211.1–829.3	6	155.3–370.5
Cocaethylene	6	12.0–57.0	6	6.6–40.4	6	15.8–53.7	6	7.9–30.4	6	14.3–45.6	6	9.1–37.4	6	9.8–22.1	6	5.8–10.6
THC	2	3.2–30.4	2	4.2–29.7	2	8.7–24.2	2	5.3–11.4	2	9.1–12.6	2	12.1–19.9	2	3.0–17.3	1	4.9
THCCOOH	6	176.7–1 354.8	6	205.6–1 048.3	6	230.7–681.2	6	172.6–653.2	6	177.5–666.9	6	276.2–2 290.0	6	247.0–630.8	6	200.0–447.3

ND: not detected; 6-MAM: 6-monoacetylmorphine; EDDP: 2-ethylidene-1,5-dimethyl-3,3-diphenylpyrrolidine; MDA: 3,4-methylenedioxyamphetamine; MDMA: 3,4-methylenedioxymethamphetamine; BE: benzoylecgonine; THC: δ-9-tetrahydrocannabinol; THCCOOH: 11-*nor*-9-carboxy-tetrahydrocannabinol.

#### Amphetamines

The compounds analysed for the amphetamines group were amphetamine, methamphetamine, MDA and MDMA. All these compounds can be consumed as drugs of abuse, and amphetamine and MDA are also metabolites of methamphetamine and MDMA, respectively. Amphetamine is as well a major component of prescription stimulants, such as Adderall^®^. Amphetamine and methamphetamine were detected in all the wastewater samples (*n* = 48). Among all the WWTPs, the highest concentration of amphetamine was 265.1 ng/mg in Newtown Creek-Manhattan before Memorial Day and the lowest was 12.9 ng/mg in Hunts Point (The Bronx) after New Year’s. Methamphetamine had the highest concentration of 340.5 ng/mg in Tallman (Queens) before New Year's and the lowest was seen in Hunts Point (The Bronx) at 15.7 ng/mg after New Year’s. MDA was detected in 22 samples and MDMA in 38 samples out of 48. MDA was not detected in any wastewater samples from Hunts Point (The Bronx), and MDMA only in one sample from that location (9.9 ng/mg). The highest concentration of MDA was 37.4 ng/mg in Jamaica (Queens) after Labour Day and the lowest concentration detected was 2.7 ng/mg in Newtown Creek-Brooklyn Queens pool before Memorial Day. MDMA had its highest concentration of 223.8 ng/mg in Tallman (Queens) after Labour Day and lowest concentration of 4.7 ng/mg in Jamaica (Queens) before New Year’s. North River (northern Manhattan) and Tallman (Queens) were both significantly higher from the other treatment plants (*F* = 6.918, *P* = 3.97 × 10^–4^), except Newtown Creek-Manhattan. No significant difference was found between holidays (*F* = 0.584, *P* = 0.631) and no interaction effect was determined (*F* = 0.429, *P* = 0.954) when looking at holiday and location together. Amphetamine, methamphetamine, MDMA and MDA results for all WWTPs and collection times are shown in Figure 2(B), and Tables 3, 4, and Supplementary Tables S1–S6.

#### Opioids

The compounds defined in this group were 6-MAM, morphine, codeine, oxymorphone, oxycodone, hydromorphone, hydrocodone, fentanyl, norfentanyl, methadone and EDDP. In decreasing concentrations order, morphine, EDDP, methadone, codeine, hydromorphone, oxymorphone and oxycodone tested positive for all samples (*n* = 48). Thirty-three samples were positive for hydrocodone and eight samples tested positive for 6-MAM. Fentanyl was detected only once and norfentanyl was not detected in any of the treatment plants.

Among all the WWTP’s, the highest concentration of morphine was 1 942.7 ng/mg in Hunts Point (The Bronx) before Memorial Day and the lowest was 82.7 ng/mg in North River (northern Manhattan) after New Year’s. Methadone’s highest concentration was 361.2 ng/mg in Hunts Point (The Bronx) before Memorial Day and for EDDP was 698.1 ng/mg also in Hunts Point (The Bronx) but after Labour Day. Both analytes showed the lowest concentration (methadone 17.3 and EDDP 36.4 ng/mg) in Jamaica (Queens) after New Year's. Codeine had its highest concentration of 321.1 ng/mg in Jamaica (Queens) after Labour Day and lowest of 31.5 ng/mg in Newtown Creek-Manhattan after Memorial Day. The highest concentration of hydromorphone was 234.2 ng/mg in Newtown Creek-Manhattan before Memorial Day and lowest of 4.1 ng/mg in Newtown Creek-Brooklyn/Queens after New Year’s. In the case of oxymorphone, the highest concentration was 166.5 ng/mg before Memorial Day in Hunts Point (The Bronx) and the lowest was 13 ng/mg after Memorial Day in Newtown Creek-Brooklyn/Queens. Oxycodone showed its highest concentration of 127.5 ng/mg in Tallman (Queens) before New Year’s and lowest of 6.7 ng/mg in Newtown Creek-Brooklyn/Queens after Memorial Day. Hydrocodone, which was detected in 33 cases, showed the highest concentration (11.6 ng/mg) in Jamaica (Queens) after Labour Day and the lowest, 1.9 ng/mg, in Newtown Creek-Brooklyn/Queens after Memorial Day. The marker of heroin consumption, 6-MAM, was detected in eight samples, mostly in The Bronx (*n* = 7), being the highest concentration 25.4 ng/mg in Hunts Point (The Bronx) after 4th of July and the lowest was seen in the same location at a concentration of 4.8 ng/mg before Labour Day. Fentanyl was detected only once in Newtown Creek-Manhattan after the 4th of July with a concentration of 5.3 ng/mg. When looking at the whole group, ANOVA showed a significant difference between the four holidays analysed (*F* = 5.016, *P* = 0.008). In particular, New Year’s was significantly lower than Memorial Day and Labour Day. In addition, a significant difference was seen between Hunts Point, which had the highest values, and the other treatment plants (*F* = 32.781, *P* = 5.66 × 10^–10^). Lastly, the interaction effect was seen when location and holiday were both considered (*F* = 3.267, *P* = 0.005); in Hunts Point during Memorial Day, 4th of July and Labour Day, there were significantly higher concentrations of opioids compared to the other treatment plants during the same holidays. 6-MAM, morphine, codeine, oxymorphone, oxycodone, hydromorphone, hydrocodone, fentanyl, norfentanyl, methadone and EDDP results for all WWTPs and collection times are shown in Figure 2(C), and Tables 3, 4 and Supplementary Tables S1–S6.

#### Cocaine

This group includes cocaine and its two metabolites, BE and cocaethylene, which is only present when cocaine is consumed with ethanol. All 48 samples were positive for these analytes. The analyte with the highest concentrations was BE, followed by cocaine then cocaethylene. BE and cocaethylene had their highest concentration, BE 3 947.2 ng/mg and cocaethylene 57.0 ng/mg, in Hunts Point (The Bronx) before Memorial Day. BE’s lowest concentration was 389.6 ng/mg in Newtown Creek-Brooklyn/Queens after Memorial Day, and for cocaethylene the lowest concentration was 5.8 ng/mg in Tallman (Queens) after New Year’s. The highest concentration of cocaine was 1 814.8 ng/mg in Hunts Point (The Bronx) before 4th of July and the lowest was 92.4 ng/mg in Newtown Creek-Brooklyn/Queens after Memorial Day. Based on the ANOVA analysis, an effect due to holiday was seen (*F* = 3.137, *P* = 0.044). In particular, drug concentrations during Labour Day were significantly higher than New Year’s. In addition, an effect due to location was observed (*F* = 28.923, *P* = 2.03 × 10^–9^). Concentrations in Hunts Point were significantly higher than the other treatment plants. An interaction effect was also observed when looking at location and holiday together (*F* = 2.335, *P* = 0.031); in Hunts Point during Memorial Day, 4th of July and Labour Day there were significantly higher concentrations of cocaine compared to the other treatment plants during the same holidays. Cocaine, BE and cocaethylene results for all WWTPs and collection times are shown in Figure 2(D), and Tables 3, 4 and Supplementary Tables S1–S6.

#### Cannabis

In this category, THC and its metabolite THCCOOH were analysed. THC was found in 15 samples from two WWTPs, which were North River, northern Manhattan (4.9–30.4 ng/mg) and Jamaica, Queens (3.0–12.0 ng/mg), while in the other locations no THC was detected. THCCOOH was detected in all wastewater samples (*n* = 48). THCCOOH was found at the highest concentration (2 290.0 ng/mg) in Hunts Point (The Bronx) after Labour Day, while the lowest concentration (172.6 ng/mg) was detected in Tallman (Queens) after 4th of July. Based on ANOVA, an effect due to location was observed (*F* = 3.820, *P* = 0.011). Drug concentrations in Hunts Point were significantly higher than all other treatment plants, except Newtown Creek-Manhattan. The main effect of holiday was non-significant (*F* = 1.206, *P* = 0.329) as was the interaction effect (*F* = 1.063, *P* = 0.434). THC and THCCOOH results for all WWTPs and collection times are shown in Figure 2(E), and Tables 3, 4 and Supplementary Tables S1–S6.

## Discussion

We developed and validated an analytical method for the determination of 21 drugs and metabolites, including nicotine, amphetamines, cocaine, opioids and cannabis markers in wastewater. This method was based on a previous publication from our group [39], but with modifications. In the current method, due to the employment of a more sensitive LC-MS/MS instrument (LCMS-8050 *vs*. LCMS-8030), the initial amount of sample could be reduced from 100 to 50 mL, facilitating the extraction procedure. The former method allowed the determination of opioids and cannabis, while in the present method an expanded opioids panel (6-MAM, methadone, EDDP, fentanyl and norfentanyl), nicotine (cotinine), amphetamines (amphetamine, methamphetamine, MDA, MDMA) and cocaine (cocaine, BE and cocaethylene) compounds could be detected, achieving a LOQ between 5 and 10 ng/L. The main analytical challenge in wastewater analysis has been the development of multi-analyte methods including analytes of different chemical properties, such as basic and relatively hydrophilic drugs, nicotine, amphetamines, cocaine and opioids markers, and acidic and lipophilic compounds, like THC and THCCOOH [38]. In the literature, there are methods for the determination of multiple drug groups, including basic drugs and cannabis, in wastewater samples [33–37]. All these methods employed LC-MS/MS. Bijlsma et al. [33] developed a method for the determination of amphetamines, cocaine and THCCOOH, employing 50 mL and achieving a LOD of 500 ng/L for THCCOOH, higher than in our method (5 ng/L). Boleda et al. [34] developed a method for the determination of opioids and cannabinoids, including THC and THCCOOH. They achieved a similar LOQ for the cannabinoids (8.3–12.5 ng/L) as in our method (5 ng/L) but they required 200 mL of water, while we extracted 50 mL, making the extraction procedure faster. Castiglioni et al. [35] developed a method for the simultaneous extraction of amphetamines, cocaine, opioids and THCCOOH from wastewater samples; however, the instrumental analysis was performed using four different chromatographic separations. In our present method we performed two different gradients, using the same column and mobile phase. Postigo et al. [36] and Tscharke et al. [37] employed two different extractions and analytical procedures for basic drugs and cannabinoids analysis; we employed one extraction procedure and two different chromatographic separations.

Regarding creatinine analysis, we developed a quick method that allowed the determination of creatinine in 200 µL of wastewater, achieving a LOQ 0.01 mg/L. Simple dilute and shoot creatinine methods in wastewater have been previously published [42–44], showing our method a similar sensitivity and specificity. The concentrations reported in our study, 0.22–2.68 mg/L, were within the range of concentrations previously reported (0.06–10.7 mg/L) [32]. Although some studies reported stability issues of creatinine in wastewater and in the wastewater system [32], it has been successfully employed by other authors in the USA [28, 29], as a normalization factor to account for population variations among sampling periods. In this study, the variability (CV) of creatinine concentrations within each wastewater plant at eight different time points was between 20% and 33.9%, except in the case of Tallman, which was 72.8%. This variability of creatinine concentrations may impact the variability of the normalized concentrations of the drugs and metabolites. It is important to highlight that creatinine is still being investigated in WBE as a population biomarker [29, 32].

In both methodologies, the multi-analyte method and the creatinine method, we employed surrogate matrices (deionized water, river water) to evaluate certain validation parameters. Wastewater sample composition is highly variable and it is often difficult to find blank samples negative for all the analytes of interest. The surrogate matrices employed were different from the complex authentic wastewater samples, and therefore, the evaluation of key validation parameters in LC-MS/MS, such as matrix effect, may be compromised. To compensate for these effects, we used as internal standards the deuterated analogues of the measured compounds, as it is recommended in the WBE field [45].

The multi-analyte and the creatinine methods were applied for the analysis of 48 authentic wastewater samples collected from six WWTPs in New York City throughout 1 year. Due to DEP safety rules and policies, only a one-time grab samples (in triplicate) from the wastewater plant primary settling pool could be collected, and this collection was performed by DEP authorized personnel. This sample collection mode is the main limitation of our study. As indicated by Ort el al. [46], a composite sampling procedure, high-frequency and flow-proportional sampling mode, employing the adequate equipment, is recommended to collect a representative 24-h composite sample and to calculate the daily load (mg/day). The daily load is calculated multiplying the concentration of the drug in 24-h composite sample by the daily flow rate of the wastewater plant, and when available, by the correction factor, which considers the mean excretion rate of the analyte and the molecular mass ratio parent drug/metabolite [47]. Due to the sample collection mode in this study, one-time grab sample, the daily load could not be calculated. We collected the samples from the primary settling pool at the same time frame (8:00 am to 11:00 am) from six different wastewater plants, and we normalized the concentrations by creatinine, in order to reduce the variability due to the difference in population served, and to be able to compare the different days and locations. In order to do an estimation of the community use, we multiplied the individual normalized concentrations by the currently available correction factors [37, 40]. Despite the sample collection limitation, this study provides the first preliminary indication by wastewater analysis of the distribution of licit and illicit drug in different boroughs of the largest city in the USA throughout 1 year.

By comparing data results of drug analysis in wastewater samples with drug consumption results from population surveys conducted nationally and regionally, it has been shown that wastewater analysis data and survey results are not directly comparable but rather complementary [8]. We compared our analytical results based on normalized concentrations, adjusted and not adjusted by correction factors, to data collected by national and local agencies. According to the results obtained in this study, the most present analyte, in terms of normalized concentration levels, was BE followed by cotinine, morphine and THCCOOH. When the normalized concentrations were adjusted by the correction factors, the most present drug was THCCOOH, followed by BE, cotinine and morphine. Based on 2016 NSDUH report, the most used drugs were tobacco, cannabis, opioids, cocaine and amphetamines in decreasing order [1]. The main limitation of our data, as previously explained, is that the sample collection mode was one-time grab instead of 24-h composite sample, and therefore the daily load adjusted by correction factors could not be performed. Because of this, a direct comparison of these sets of data is difficult to perform. When looking at other studies performed in the USA, it was possible to make a comparison between rankings of the most present drugs analysed. The Pacific Northwest region showed higher concentrations of amphetamine, followed by BE and cocaine [29]. Similarly, in the Midwestern region, amphetamine and methamphetamine also presented the highest mass loadings, approximately twofold higher than the reported BE mass loadings from the same area [30]. As the authors suggested, these differences highlight the various use patterns of illicit drugs. In fact, when compared to the results of the present study, it is interesting to see how cocaine prevailed in New York City while amphetamines were ranked fairly lower. To corroborate this inference, a study in upstate New York presented interesting results. Subedi and Kannan [48] showed that in this region BE had the highest concentrations in influent wastewater from two different wastewater plants. These values were followed by opioids and methamphetamines. Similarly, this study presented the same concentration order, underlining that New York may have a higher use of cocaine compared to other regions of the USA. The Drug Enforcement Administration (DEA) has noted a resurgence of cocaine in the past years, and New York is one of the major states with high cocaine availability [49].

Comparing the drug concentrations among the different boroughs, cocaine and opioids (heroin, prescription opioids, methadone) were mostly present in The Bronx. A local survey on drug overdose deaths (OD) that was conducted by New York City Health Department, reported that The Bronx had the largest number of OD, and these ODs in New York City were due to heroin (55%), followed by cocaine (46%), opioid analgesics excluding fentanyl (18%) and methadone (14%) [50]. From our data, it is highlighted how The Bronx was the only borough where 6-MAM was detected in wastewater, with the exception of after Labour Day in lower Queens. Although fentanyl was involved in half of the ODs involving heroin, it was only detected in one wastewater sample and its metabolite norfentanyl was not detected in any of them. The absence of fentanyl and norfentanyl in the samples could be attributed to the small doses of fentanyl that are taken with respect to other drugs (µg doses *vs*. mg doses).

## Conclusion

We developed two sensitive and specific methods, one for the determination of nicotine, amphetamines, opioids, cocaine and cannabis, and another for the determination of creatinine in wastewater samples. We applied these methods for the analysis of wastewater samples collected from six WWTPs in New York City throughout 1 year. Amphetamines showed the highest concentrations in Manhattan and Queens, cannabis in The Bronx and Manhattan, and nicotine, cocaine and opioids were mostly present in The Bronx. After adjusting by correction factors, the most present drugs were cannabis (THCCOOH), followed by cocaine (BE), nicotine (cotinine) and opioids (morphine). Wastewater analysis showed the ability to be used as a method to identify drug use within large communities.

## Supplementary Material

Supplemental Material
