# Supplementary material for: Wastewater analysis for nicotine, cocaine, amphetamines, opioids and cannabis in New York City
Source: Forensic Sci Res. 2019 May 31;4(2):152–67. doi: 10.1080/20961790.2019.1609388 (PMC6609350; doi:10.1080/20961790.2019.1609388)
Supplement: Supplemental Material [file TFSR_A_1609388_SM1122.docx]

Table S1. Raw concentrations in ng/L at each collection day in the wastewater treatment plant Hunts Point (The Bronx).

| Analytes | 2016/17 Collection Day | | | | | | | |
| --- | --- | --- | --- | --- | --- | --- | --- | --- |
|  | Memorial Day | | 4th July | | Labor Day | | New Year's Day | |
|  | May 27 | May 31 | July 1 | July 5 | September 2 | September 6 | December 30 | January 3 |
| Cotinine | **1 467.1** | 1 350.2 | 1 373.1 | *532.7* | 923.5 | 1 031.8 | 590.7 | 700.4 |
| Morphine | **1 373.5** | 1 280.8 | 1 096.0 | *237.9* | 918.6 | 911.0 | 421.4 | 562.1 |
| Oxymorphone | **117.7** | 103.6 | 106.0 | *35.7* | 59.2 | 87.6 | 83.5 | 67.2 |
| Hydromorphone | 44.7 | 53.4 | 45.7 | *29* | 47.7 | **68.6** | 37.6 | 56.2 |
| Codeine | **208.3** | 174.7 | 171.7 | *44.6* | 133.5 | 145.4 | 65.2 | 151.7 |
| Amphetamine | 62.0 | **77.3** | 43 | 15.5 | 21.0 | 31.7 | *15.0* | 19.6 |
| MDA | ND | ND | ND | ND | ND | ND | ND | ND |
| Methamphetamine | 51.6 | **223.1** | 59.1 | 27.3 | 43.9 | 56.3 | 53.8 | *21.6* |
| Oxycodone | 75.8 | 60.7 | **80.5** | *21.3* | 33.4 | 48.1 | 30.8 | 33.8 |
| 6-MAM | ND | 17.0 | 6.9 | 12.0 | *3.6* | 4.5 | **18.8** | 7.1 |
| Hydrocodone | 5.0 | **7.8** | 5.4 | ND | ND | *4.4* | ND | ND |
| MDMA | **7.0** | ND | ND | ND | ND | ND | ND | ND |
| Norfentanyl | ND | ND | ND | ND | ND | ND | ND | ND |
| BE | 2 790.7 | **2 811.1** | 2 487.8 | *1 195.0* | 2 456.0 | 2 121.9 | 2 014.3 | 1 367.2 |
| Cocaine | 1 140.7 | 1 031.7 | **1 564.4** | *360.0* | 787.1 | 708.9 | 788.6 | 509.1 |
| Cocaethylene | 40.3 | 36.9 | **46.3** | *14.4* | 34.5 | 23.7 | 25.3 | 14.5 |
| Fentanyl | ND | ND | ND | ND | ND | ND | ND | ND |
| EDDP | 463.7 | **532.3** | 481.0 | *187.3* | 381.9 | 442.6 | 267.2 | 241.9 |
| Methadone | 255.4 | **270.3** | 250.4 | *91.2* | 195.8 | 205.3 | 157.4 | 125.4 |
| THC | ND | ND | ND | ND | ND | ND | ND | ND |
| THCCOOH | 597.8 | **957.1** | 587.2 | *309.0* | 504.2 | 1 451.9 | 459.4 | 614.6 |

Bold values are maximum concentrations, while italics are the minimum concentration of the analyte. ND: not detected.

Table S2. Raw concentrations in ng/L at each collection day in the wastewater treatment plant North River (northern Manhattan).

| Analytes | 2016/17 Collection Day | | | | | | | |
| --- | --- | --- | --- | --- | --- | --- | --- | --- |
|  | Memorial Day | | 4th July | | Labor Day | | New Year's Day | |
|  | May 27 | May 31 | July 1 | July 5 | September 2 | September 6 | December 30 | January 3 |
| Cotinine | 427.1 | *340.5* | 426.0 | 384.6 | 452.8 | **475.4** | 360.9 | 401.9 |
| Morphine | 205.0 | 203.9 | 281.3 | 222.7 | 336.0 | **431.0** | 322.6 | *159.5* |
| Oxymorphone | 38.4 | 38.1 | 34.8 | *26.0* | 45.8 | 41.0 | **51.2** | 29.5 |
| Hydromorphone | 8.4 | 6.8 | 11.9 | *6.2* | **60.8** | 8.8 | 12.9 | 10.4 |
| Codeine | 99.6 | *60.1* | 90.7 | 64.4 | 79.7 | **102.6** | 76.1 | 65.9 |
| Amphetamine | 155.9 | 138.2 | 167.2 | *103.2* | **203.7** | 149.7 | 167.0 | 146.2 |
| MDA | ND | 14.8 | ND | 6.2 | ND | 24.6 | **51.1** | 11.1 |
| Methamphetamine | 227.1 | 342.1 | 272.3 | *218.0* | 367.8 | 292.0 | **372.2** | 313.5 |
| Oxycodone | 24.7 | 28.3 | 30.2 | 23.0 | **32.3** | 26.6 | 28.2 | *15.1* |
| 6-MAM | ND | ND | ND | ND | ND | ND | ND | ND |
| Hydrocodone | 7.0 | 5.7 | 6.1 | **8.9** | 6.0 | 6.8 | 6.0 | 5.8 |
| MDMA | 5.6 | 39.1 | 10.7 | 20.3 | 14.7 | 83.2 | **187.0** | 47.5 |
| Norfentanyl | ND | ND | ND | ND | ND | ND | ND | ND |
| BE | 1 031.1 | *697.4* | 1174.9 | 931 | **1 276.5** | 833.8 | 1 260.5 | 928.7 |
| Cocaine | 499.8 | 269.7 | 608.2 | 366.3 | 383.0 | *239.7* | **667.6** | 313.1 |
| Cocaethylene | 20.1 | *10.6* | 27.8 | 19.7 | 27.5 | 11.5 | **42.6** | 18.2 |
| Fentanyl | ND | ND | ND | ND | ND | ND | ND | ND |
| EDDP | 135.3 | *106.1* | 124.6 | 129.3 | 150.1 | 144.0 | **197.9** | 107.9 |
| Methadone | 53.2 | *42.3* | 51.9 | 46.4 | **66.3** | 48.6 | 58.1 | 45.2 |
| THC | 28.9 | 32.8 | 23.2 | 16.7 | 21.3 | 24.1 | **33.3** | 9.5 |
| THCCOOH | *392.0* | **631.4** | 421.3 | 409.4 | 406.4 | 549.6 | 574.4 | 436.8 |

Bold values are maximum concentrations, while italics are the minimum concentration of the analyte. ND: not detected.

Table S3. Raw concentrations in ng/L at each collection day in the wastewater treatment plant Tallman (Queens).

| Analytes | 2016/17 Collection Day | | | | | | | |
| --- | --- | --- | --- | --- | --- | --- | --- | --- |
|  | Memorial Day | | 4th July | | Labor Day | | New Year's Day | |
|  | May 27 | May 31 | July 1 | July 5 | September 2 | September 6 | December 30 | January 3 |
| Cotinine | 771.9 | 533.3 | **805.6** | *306.8* | 525.3 | 565.3 | 450.2 | 762.8 |
| Morphine | 308.1 | 177.4 | 300.0 | 148.3 | *145.8* | 190.5 | 149.8 | **457.2** |
| Oxymorphone | **97.3** | 38.1 | 64.8 | *13.0* | 25.6 | 44.0 | 24.1 | 53.3 |
| Hydromorphone | 8.2 | 6.6 | 9.2 | *4.0* | 5.1 | 6.4 | 4.6 | **10.3** |
| Codeine | 81.3 | 21.9 | **94.1** | *20.8* | 27.8 | 47.8 | 29.3 | 87.5 |
| Amphetamine | **87.9** | 37.0 | 81.4 | *19.9* | 26.5 | 55.4 | 27.9 | 43.7 |
| MDA | ND | ND | ND | 5.1 | ND | **8.4** | ND | ND |
| Methamphetamine | **195.3** | 92.0 | 187.3 | 90.0 | 79.7 | 166.3 | 75.6 | *49.1* |
| Oxycodone | **75.8** | 30.6 | 55.2 | *13.0* | 21.4 | 44.8 | 28.3 | 39.6 |
| 6-MAM | ND | ND | ND | ND | ND | ND | ND | ND |
| Hydrocodone | **7.8** | ND | 7.2 | ND | ND | 4.5 | ND | ND |
| MDMA | 62.0 | 81.7 | 36.7 | 24.5 | 22.4 | **116.8** | 37.2 | *18.7* |
| Norfentanyl | ND | ND | ND | ND | ND | ND | ND | ND |
| BE | 743.7 | 459.4 | 743.1 | *266.4* | 517.2 | 625.1 | 473.5 | **1241.3** |
| Cocaine | 349.5 | 141.7 | **369.1** | *76.8* | 243.4 | 280.8 | 184.1 | 314.1 |
| Cocaethylene | **12.7** | 4.6 | 11.0 | *3.1* | 6.9 | 5.6 | 4.7 | 11.2 |
| Fentanyl | ND | ND | ND | ND | ND | ND | ND | ND |
| EDDP | 54.7 | 32.0 | 54.3 | *23.6* | *23.6* | 32.1 | 26.8 | **123.6** |
| Methadone | 20.9 | 12.8 | 31.6 | *9.2* | 13.6 | 15.5 | 12.1 | **54.6** |
| THC | ND | ND | ND | ND | ND | ND | ND | ND |
| THCCOOH | 187.5 | 134.2 | 228.7 | *68* | 85.6 | 144.2 | 140.0 | **606.0** |

Bold values are maximum concentrations, while italics are the minimum concentration of the analyte. ND: not detected.

Table S4. Raw concentrations in ng/L at each collection day in the wastewater treatment plant Jamaica (Queens).

| Analytes | 2016/17 Collection Day | | | | | | | |
| --- | --- | --- | --- | --- | --- | --- | --- | --- |
|  | Memorial Day | | 4th July | | Labor Day | | New Year's Day | |
|  | May 27 | May 31 | July 1 | July 5 | September 2 | September 6 | December 30 | January 3 |
| Cotinine | 711.8 | 634.3 | 726.1 | 551.7 | 731.7 | **827.9** | 657.0 | *363.2* |
| Morphine | 430.7 | 303.8 | 499 | 278.9 | 425.3 | **515.7** | 408.1 | *225.7* |
| Oxymorphone | 67.5 | 43.0 | 62.9 | 38.3 | **74.4** | 52.3 | 60.5 | *30.9* |
| Hydromorphone | 10.1 | 10.6 | **16.8** | 8.2 | 14.2 | 9.1 | 14.3 | *7.5* |
| Codeine | 83.2 | 90.0 | 87.7 | 64.3 | 102.8 | **219.6** | 74.3 | *34.1* |
| Amphetamine | 41.3 | 62.0 | **71.6** | 29.4 | 44.0 | 67.5 | *23.6* | 36.9 |
| MDA | ND | ND | ND | 5.9 | ND | **25.6** | ND | 6.8 |
| Methamphetamine | *28.6* | 42.5 | 80.9 | 30.3 | 52.2 | 36.4 | 46.5 | **132.6** |
| Oxycodone | 33.9 | 35.7 | **37.9** | *22.1* | 33 | 34.8 | 36.1 | 27.7 |
| 6-MAM | ND | ND | ND | ND | ND | **6.4** | ND | ND |
| Hydrocodone | 5.9 | 6.2 | 7.8 | 4.6 | 7.8 | **7.9** | 4.9 | ND |
| MDMA | ND | 15.6 | ND | 13.8 | ND | **74.8** | 7.8 | 73.9 |
| Norfentanyl | ND | ND | ND | ND | ND | ND | ND | ND |
| BE | 1 299.7 | 1 139.1 | 1 159.1 | 811.8 | **1 402.4** | 1 142.7 | 1 213.2 | *530.4* |
| Cocaine | **638.2** | 347.6 | 470.2 | *199.9* | 522.8 | 321.8 | 398.3 | 243.6 |
| Cocaethylene | **20.2** | 14.1 | 16.5 | 9.8 | 17.4 | 11.9 | 16.3 | *6.4* |
| Fentanyl | ND | ND | ND | ND | ND | ND | ND | ND |
| EDDP | 91.8 | 97.8 | 87.8 | 70 | 71.9 | 90.1 | **101.2** | *29.1* |
| Methadone | **60.7** | 59.9 | 55.6 | 39.2 | 50.9 | 47.3 | 59.4 | *13.8* |
| THC | 5.2 | 6.4 | 9.1 | 4.7 | **9.7** | 8.2 | 5.0 | ND |
| THCCOOH | **523.1** | 327 | 241.3 | 315.2 | 439.4 | 444.7 | 410.8 | *160* |

Bold values are maximum concentrations, while italics are the minimum concentration of the analyte. ND: not detected.

Table S5. Raw concentrations in ng/L at each collection day in the wastewater treatment plant Newtown Creek-Brooklyn/Queens.

| Analytes | 2016/17 Collection Day | | | | | | | |
| --- | --- | --- | --- | --- | --- | --- | --- | --- |
|  | Memorial Day | | 4th July | | Labor Day | | New Year's Day | |
|  | May 27 | May 31 | July 1 | July 5 | September 2 | September 6 | December 30 | January 3 |
| Cotinine | 764.6 | 665.3 | 803.5 | *658.5* | 757.1 | **1 350.9** | 776.1 | 773.1 |
| Morphine | 608.0 | 568.3 | **821.4** | *501.8* | 631.1 | 706.1 | 578.5 | 531.5 |
| Oxymorphone | 28.5 | 35.1 | **38.4** | *23.1* | 29.8 | 28.8 | 24.1 | 30.9 |
| Hydromorphone | 13.2 | 11.3 | 14.7 | 12.1 | 9.3 | **18.6** | 10.8 | *8.6* |
| Codeine | **153.1** | 120.4 | 119.3 | 80.3 | 109.6 | 126.4 | *69.5* | 88.6 |
| Amphetamine | **171.4** | 113.5 | 170.3 | 131.9 | 136.7 | 137.9 | 104.3 | *90.2* |
| MDA | 4.2 | 9.7 | 6.5 | **35.3** | ND | 14.3 | 28.8 | 22.9 |
| Methamphetamine | **221.0** | 66.6 | 98.1 | 78.8 | *55.7* | 80.7 | 58.9 | 64.5 |
| Oxycodone | 19.4 | 17.9 | **26.7** | 16.0 | 21.5 | 16.2 | *14.6* | 17.5 |
| 6-MAM | ND | ND | ND | ND | ND | ND | ND | ND |
| Hydrocodone | 5.7 | 5.0 | **6.9** | 5.0 | 4.7 | 5.7 | ND | ND |
| MDMA | *10.3* | 29.1 | 19.8 | 69.0 | 33.8 | 51.4 | **71.3** | 53.2 |
| Norfentanyl | ND | ND | ND | ND | ND | ND | ND | ND |
| BE | 1 734.4 | *1 044.9* | 1 943.8 | 1 599 | **2 191.5** | 1 661.3 | 1 734.9 | 1 406.3 |
| Cocaine | 337.1 | *247.8* | **641.2** | 261.7 | 605.4 | 329.1 | 368.1 | 327.9 |
| Cocaethylene | 34.4 | *17.7* | **43.7** | 29.6 | 36.3 | 23.8 | 34.7 | 17.8 |
| Fentanyl | ND | ND | ND | ND | ND | ND | ND | ND |
| EDDP | 308.8 | 228.3 | **311.8** | *198.5* | 239.4 | 277.1 | 225.9 | 211.1 |
| Methadone | 148.7 | 120.6 | **171.1** | 101.0 | 130.2 | 124.7 | 118.3 | *88.6* |
| THC | ND | NA | ND | ND | ND | ND | ND | ND |
| THCCOOH | 848.1 | NA | 596.1 | *499.7* | 668.9 | **929.8** | 693.7 | 898 |

Bold values are maximum concentrations, while italics are the minimum concentration of the analyte. NA: not available; ND: not detected.

Table S6. Raw concentrations in ng/L at each collection day in the wastewater treatment plant Newtown Creek-Manhattan.

| Analytes | 2016/17 Collection Day | | | | | | | |
| --- | --- | --- | --- | --- | --- | --- | --- | --- |
|  | Memorial Day | | 4th July | | Labor Day | | New Year's Day | |
|  | May 27 | May 31 | July 1 | July 5 | September 2 | September 6 | December 30 | January 3 |
| Cotinine | **443.9** | *276* | 430.7 | 325.1 | 344.9 | 375.4 | 370.5 | 336.4 |
| Morphine | 287.5 | **297.2** | 247.7 | *197.6* | 227.2 | 269.7 | 250 | 228.6 |
| Oxymorphone | 47.3 | *30.4* | **53.8** | *30.4* | 32.3 | 31.5 | 48.2 | 34.2 |
| Hydromorphone | **209.8** | *7.1* | 14.8 | 8.5 | 14.4 | 14.1 | 12.1 | 9.5 |
| Codeine | 53.2 | *43.4* | **93.6** | 58.3 | 53.0 | 71.6 | 78.3 | 67.6 |
| Amphetamine | 237.5 | 156.4 | **285.9** | 173.4 | 213.1 | *136.4* | 167.2 | 160.5 |
| MDA | ND | 8.3 | ND | 6.7 | ND | 12.3 | **20.7** | 9.6 |
| Methamphetamine | 98.8 | 109.8 | **154.9** | 88.3 | 138.7 | *80.5* | 93.2 | 125.1 |
| Oxycodone | 30.1 | 24.2 | **40.9** | 23.3 | 25.2 | *18.6* | 32.6 | 22.8 |
| 6-MAM | ND | ND | ND | ND | ND | ND | ND | ND |
| Hydrocodone | 5.0 | 4.6 | ND | 4.3 | ND | ND | 4.6 | **14.5** |
| MDMA | 10.9 | 23.6 | 14.5 | 31.3 | 9.3 | 51.3 | **80.1** | 35.1 |
| Norfentanyl | ND | ND | ND | ND | ND | ND | ND | ND |
| BE | 985.9 | *647.4* | **1 114.5** | 669.6 | 966.3 | 684.1 | 1 055.6 | 744.7 |
| Cocaine | 409.5 | *199.2* | **489.2** | 211.3 | 353.7 | 221.7 | 425.6 | 275.3 |
| Cocaethylene | 26.4 | 10.5 | **33.0** | 12.4 | 21.9 | *9.7* | 31.7 | 10.4 |
| Fentanyl | ND | ND | ND | **5.9** | ND | ND | ND | ND |
| EDDP | 122.0 | 145.6 | 136.4 | 126.2 | *114.1* | 140.6 | **161.3** | 121.3 |
| Methadone | **73.3** | *44.1* | 56.9 | 47.9 | 48.6 | 49.2 | 63.6 | 50.1 |
| THC | ND | ND | ND | ND | ND | ND | ND | ND |
| THCCOOH | **1 213.9** | 410.3 | *317.8* | 626.2 | 353.6 | 430.5 | 475.0 | 472.9 |

Bold values are maximum concentrations, while italics are the minimum concentration of the analyte. ND: not detected.
